# Artificial Intelligence–Enhanced OCT Biomarkers Analysis in Macula-off Rhegmatogenous Retinal Detachment Patients

**DOI:** 10.1167/tvst.13.10.21

**Published:** 2024-10-11

**Authors:** Lorenzo Ferro Desideri, Tamara Danilovska, Enrico Bernardi, Dmitri Artemiev, Karin Paschon, Michel Hayoz, Alain Jungo, Raphael Sznitman, Martin S. Zinkernagel, Rodrigo Anguita

**Affiliations:** 1 Department of Ophthalmology, Inselspital, Bern University Hospital, University of Bern, Bern, Switzerland; 2 Department for BioMedical Research, University of Bern, Bern, Switzerland; 3 Bern Photographic Reading Center, Inselspital, Bern University Hospital, University of Bern, Bern, Switzerland; 4 ARTORG Research Center Biomedical Engineering Research, University of Bern, Bern, Switzerland; 5 Moorfields Eye Hospital NHS Foundation Trust, London, UK

**Keywords:** retinal detachment, artificial intelligence, macula off, artificial intelligence in retina, biomarkers, gas tamponade, vitrectomy, surgery, OCT

## Abstract

**Purpose:**

To identify optical coherence tomography (OCT) biomarkers for macula-off rhegmatogenous retinal detachment (RRD) with artificial intelligence (AI) and to correlate these biomarkers with functional outcomes.

**Methods:**

Patients with macula-off RRD treated with single vitrectomy and gas tamponade were included. OCT volumes, taken at 4 to 6 weeks and 1 year postoperative, were uploaded on an AI-derived platform (Discovery OCT Biomarker Detector; RetinAI AG, Bern, Switzerland), measuring different retinal layer thicknesses, including outer nuclear layer (ONL), photoreceptor and retinal pigmented epithelium (PR + RPE), intraretinal fluid (IRF), subretinal fluid, and biomarker probability detection, including hyperreflective foci (HF). A random forest model assessed the predictive factors for final best-corrected visual acuity (BCVA).

**Results:**

Fifty-nine patients (42 male, 17 female) were enrolled. Baseline BCVA was 0.5 logarithmic minimum angle of resolution (logMAR) ± 0.1, significantly improving to 0.3 ± 0.1 logMAR at the final visit (*P* < 0.001). Average thickness analysis indicated a significant increase after the last follow-up visit for ONL (from 95.16 ± 5.47 µm to 100.8 ± 5.27 µm, *P* = 0.0007) and PR + RPE thicknesses (60.9 ± 2.6 µm to 66.2 ± 1.8 µm, *P* = 0.0001). Average occurrence rate of HF was 0.12 ± 0.06 at initial visit and 0.08 ± 0.05 at last follow-up visit (*P* = 0.0093). Random forest model revealed baseline BCVA as the most critical predictor for final BCVA, followed by ONL thickness, HF, and IRF presence at the initial visit.

**Conclusions:**

Increased ONL and PR-RPE thickness associate with better outcomes, while HF presence indicates poorer results, with initial BCVA remaining a primary visual predictor.

**Translational Relevance:**

The study underscores the role of novel biomarkers like HF in understanding visual function in macula-off RRD.

## Introduction

Despite the remarkable progress achieved in the past years in the surgical management of rhegmatogenous retinal detachment (RRD), patients, particularly those with macula-off RRDs, can still face suboptimal postoperative visual outcomes.^1^^,^^2^ Consistent evidence has been provided regarding the long-term visual impairment in association with the detachment of the neurosensory retina from underlying retinal pigment epithelium (RPE) in these patients.^3^^,^^4^

Experimental studies have shown that in a macula-off detached retina, there is a degeneration of the rod and cone outer segments, redistribution of opsins to the photoreceptor cell bodies, withdrawal of rod synaptic terminals toward their cell bodies, outgrowth of rod bipolar and horizontal-cell neurites, and Müller cell proliferation and hypertrophy^4^^–^^7^; therefore, the aim of RRD repair is to promptly restore both the physiological activity and structure of the detached photoreceptors.

Multiple imaging studies have focused on identifying and describing the clinicopathological characteristics in relation to functional outcomes in successfully repaired macula-off RRDs.^8^ In this regard, several well-characterized optical coherence tomography (OCT) biomarkers have been demonstrated to be linked to the visual prognosis in these patients, including the ellipsoid zone (EZ)–RPE thickness, the EZ disruption, and the external limiting membrane (ELM).^9^^–^^11^ A recent study on en face OCT analyzing the EZ of patients operated for macula-off RRD showed that visual recovery may occur also after many years.^12^

Currently, artificial intelligence (AI) is being investigated as a powerful technological tool in the prediction of the clinical course of several retinal diseases.^13^^–^^16^ A recent study evaluated an automated deep learning (DL) model to predict the anatomic result after RRD surgery and found that the model was effective in predicting the anatomic outcomes in these patients.^17^ Despite the increasing interest in using AI to examine OCT biomarkers as a predictive tool in medical retina, no clinical study has investigated its possible application in vitreoretinal surgery.

In this research, we assessed the effectiveness of AI-based software in identifying OCT biomarkers in patients who underwent surgery for macula-off RRD. Additionally, we utilized machine learning algorithms to correlate these biomarkers with the prediction of postoperative visual acuity outcomes.

## Methods

### Study Subjects and Design

In this retrospective clinical series, patients diagnosed with macula-off RRD underwent single vitrectomy and gas at the Department of Ophthalmology at Inselspital, Bern University Hospital, Switzerland, between January 2016 and December 2022. The inclusion criteria were successful single-surgery repaired macula-off RRD with either pars plana vitrectomy alone or vitrectomy in combination with encircling band. RRD was classified as macula-off when fundus examination and OCT images showed that the detachment involved the fovea, resulting in any loss of central visual acuity.

In addition, OCT follow-up at 4 to 6 weeks and 1 year was required. Exclusion criteria were presence of other retinal and macular diseases, traumatic retinal detachment, proliferative vitreoretinopathy at presentation or subsequent development, macula-on RRDs, previous retinal surgery and need for secondary surgery due to primary surgical failure, and positive family history of systemic diseases that could affect macular status. Patients with poor image quality at both the initial and final follow-up visits due to media opacity, including cataract formation, were excluded from the study.

Demographic and clinical features were collected for each patient. Best-corrected visual acuity (BCVA) was expressed as the logarithm of the minimum angle resolution (logMAR) and collected postoperatively within 1 month from surgery and at last follow-up visit. Patients were divided into two subgroups based on their BCVA changes after the final visit. The improvement group had a decrease in BCVA of greater than 0.2 logMAR, while the neutral group had no improvement or a decrease of less than 0.2 logMAR.

Institutional review board approval was obtained, and informed consent was secured from all patients involved in the study. This retrospective study adhered to the tenets of the Declaration of Helsinki.

### OCT Imaging

**Figure 1.**
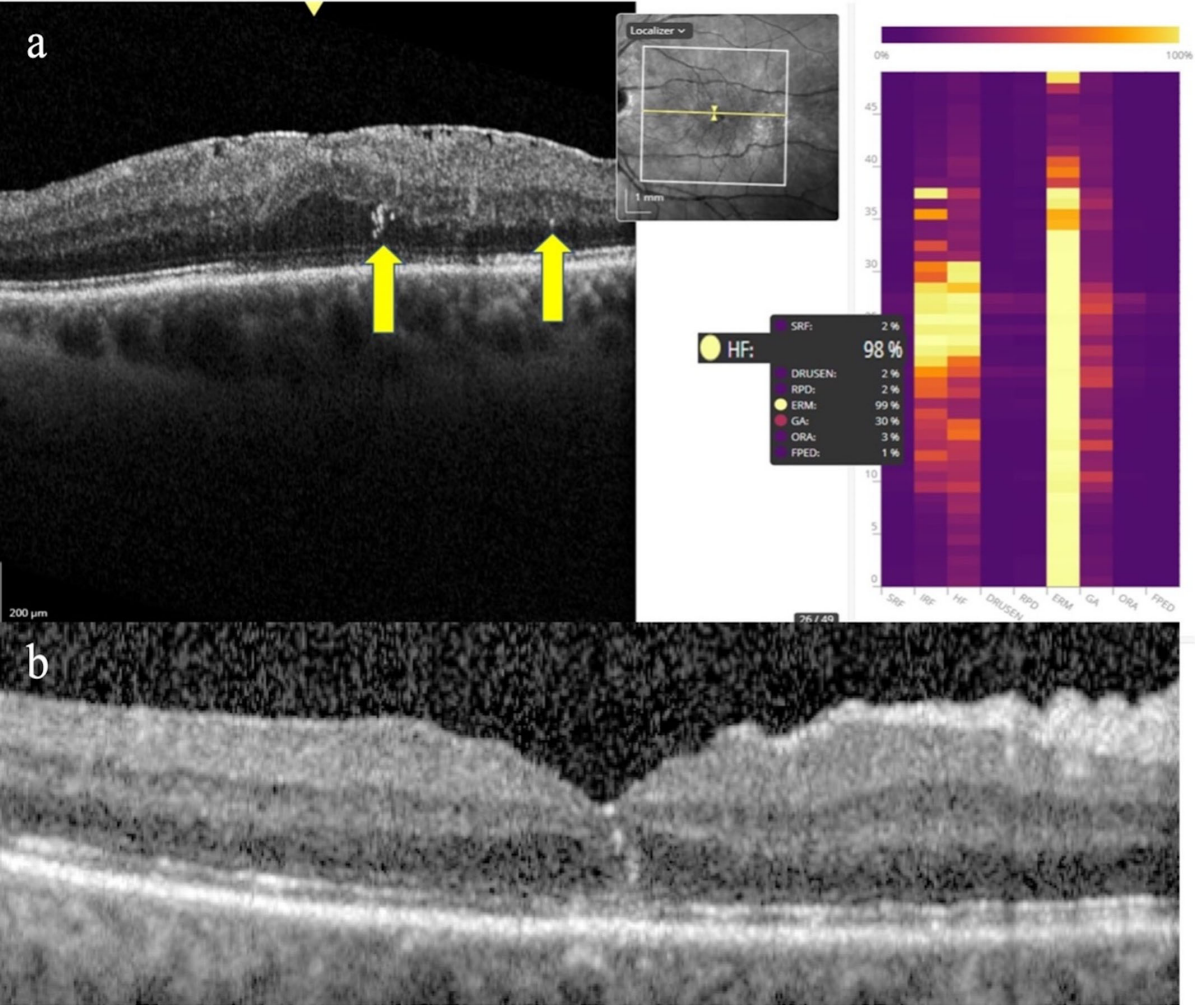
(**a**) Automated detection of biomarkers provided by Discovery. In this OCT slab, the software detects the presence of HF (*yellow arrows*) with a very high probability (98%). (**b**) Another example of a patient operated for retinal detachment with vitrectomy and gas tamponade. After 1 month, the presence of two HF in the foveal region is evident.

We used the Spectralis SD-OCT imaging system (Heidelberg Engineering, Heidelberg, Germany). OCT volumes covering an area of 5.90 × 5.75 × 1.92 mm centered on the fovea with a 49-B scan acquisition protocol and a resolution of 496 × 512 pixels per B-scan were examined. Forty-nine B-scans were averaged for each horizontal scan. OCT scans were taken at 4 to 6 weeks postoperatively and at the last follow-up visit (between 6 and 12 months) (Fig. 1).

### Quantitative Analysis: AI-Based Biomarker OCT Detector

To perform OCT images analysis, we used the AI-based platform Discovery OCT Biomarker Detector (RetinAI AG, Bern, Switzerland), which provides an automatic segmentation of retinal and choroidal thicknesses and retinal volumes/fluids. The following retinal and choroidal layers were segmented by the software: the retinal nerve fiber layer (RNFL), ganglion cell layer and inner plexiform layer (GCL + IPL), inner nuclear layer and outer plexiform layer (INL + OPL), outer nuclear layer (ONL), photoreceptor and RPE layers (PR + RPE), choriocapillaris with choroidal stroma (CC + CS), and the overall retina thickness (RT). Among the volumes, a quantitative measurement of subretinal fluid (SRF), intraretinal fluid (IRF), and pigment epithelial detachment (PED) can be obtained by the software. To confirm that there were no errors in the automatic segmentation, one of the authors (LFD) reviewed a random sample (30%). Retinal layers and volumes were calculated in the foveal region (1 mm) in the early treatment diabetic retinopathy study (ETDRS) grid. The software also predicts the presence of the following biomarkers in the macular region (6 mm) of the ETDRS grid: SRF, IRF, hyperreflective foci (HF), and epiretinal membrane (ERM) (Table 1). For each biomarker in each B-scan, the biomarker detection provides probabilities (a number ranging from 0% to 100%). Biomarkers were treated as binary variables, with probabilities above 50% considered indicative of their presence and probabilities below 50% considered indicative of their absence. We used a threshold of 50% to determine the clinical relevance of a biomarker based on the precision-recall criterion for imaging biomarker detection established by Kurmann et al.,^18^ which allowed the validation of the algorithm adopted by the software by using a convolutional neural network (CNN). For each OCT biomarker, the occurrence rate was calculated for each eye by dividing the number of B-scan biomarker presence (probability above 50%) by the total B-scans. OCT measurements of thicknesses, volumes, and biomarker probability were therefore compared between first and last follow-up visits and correlated in a multivariate analysis with last follow-up BCVA.

### Statistical Analysis

Statistical analysis was performed with SciPy (version 1.11.1), the Python package for scientific computing. The normality of the data was tested by using the Shapiro–Wilk test. The differences between sample means of biomarkers between first and last follow-up visits were tested for significance by means of a paired sample *t*-test, and correlation analysis between OCT features and final BCVA (Pearson correlation coefficient) was performed. A *P* value < 0.05 was considered statistically significant. Univariate and multivariate analyses for the prediction of final BCVA were done. A random forest model was employed to investigate the correlation between baseline demographic, clinical, and imaging postoperative features with the aim of predicting the features associated with final visual outcome at 12 months. The model's performance was assessed using metrics such as *r*-squared (*r*^2^) to evaluate the goodness of fit, and feature importance scores (FISs) were analyzed to identify the relative contributions of individual features to the predictive capacity. A 10-fold cross-validation process was used to evaluate the performance of our model.

## Results

### Demographic and Clinical Features

In total, 346 patients with macula-off RRD operated in our department were screened; among them, 59 patients were considered eligible for the study, with 71% (*n* = 42) male and 29% (*n* = 17) female. Mean ± SD age in the study population was 58.7 ± 14.2 years. Right eyes were 49% (*n* = 29) and left 51% (*n* = 30). Of the patients, 64% (*n* = 38) were phakic, whereas 21% (*n* = 21) were pseudophakic. High myopia, defined as a refractive error of ≥6 D, was reported in 10% of the subjects (*n* = 6), but none had myopic maculopathy. Fifteen percent (*n* = 9) received vitrectomy alone, while 85% (*n* = 50) had a combination of vitrectomy and encirclement surgery. The average follow-up period was 11.3 ± 1.2 months. At 4 to 6 weeks postoperatively, average BCVA was 0.45 ± 0.09 logMAR (0.4 Snellen) and last visit significantly improved to 0.29 ± 0.16 logMAR (0.5 Snellen), *P* < 0.0001. In two phakic patients, cataract surgery was needed at 6 months due to the development of clinically relevant cataract. Complete demographic and clinical features are summarized in Table 2.

**Table 1.** OCT Biomarker Definition Adopted by Discovery Software

Biomarker	Definition
SRF	Well-defined darkening with a minimal horizontal extension of 100 µm between the RPE layer and photoreceptor layer.
IRF	Diffuse darkening and thickening of the neurosensory retina and oval well-defined hyporeflective areas with a minimal extension of 25 µm in any direction between the internal limited membrane and the photoreceptor layer.
HF	Small points of increased reflectivity scattered throughout all retinal layers, primarily found in the near vicinity of intraretinal cystoid spaces. The size of HF can vary from 25 to 50 µm in diameter, and they can be clustered.
ERM	Thickening of the surface of the RNFL within the whole macular cube scan. A hyperreflective line between the vitreomacular interface and the RNFL is visible.

ERM, epiretinal membrane; HF, hyperreflective foci; IRF, intraretinal fluid; SRF, subretinal fuid.

**Table 2.** Demographic and Clinical Features of the Study Population

Demographic and/or Clinical Feature	Number (%) of Patients
Laterality	
Right	29 (49)
Left	30 (51)
SRF	10 (16)
Macular edema	13 (22)
Gender	
Male	42 (71)
Female	17 (29)
General status	
Healthy	51 (86)
Lens status	
Phakic	38 (64)
Pseudophakic	21 (36)
High myopia	6 (10)
Macula status	
2	59 (100)
Reoperations	0
Surgery type	
Vitrectomy	9 (15)
Vitrectomy + cerclage	50 (85)
Proliferative vitreoretinopathy	0
Tamponade	
Gas	59 (100)
BCVA (logMAR) 1–5 days after surgery	
2.3	46 (78)
1.9	13 (22)

### OCT Thicknesses and Volumes

Average thickness analysis showed that there was a significant increase of ONL thickness from first postoperative visit at 1 month to last follow-up visit at 12 months (from 95.16 µm ± 5.47 to 100.8 µm ± 5.27, *P* = 0.0007) and PR + RPE layer (61.72 ± 2.04 µm to 65.60 ± 1.98 µm, *P* = 0.0001). Average GLC + IPL thickness changed from 62.53 ± 6.39 µm to 62.58 ± 6.55 µm, with no significant differences (*P* = 0.9772). Average CC + CS increased from 203.70 ± 13.66 µm to 209.68 ± 16.88 µm, with nonsignificant differences (*P* = 0.1218). No significant differences were also found in the other average retinal thicknesses, including RNFL, INL + OPL, and overall RT.

In the subgroup with improved BCVA, the mean ONL thickness improved from 93.71 ± 6.42 µm to 99.99 ± 6.3 µm (*P* = 0.0022) and PR + RPE thickness from 61.36 ± 2.60 µm to 66.42 ± 2.41 µm (*P* = 0.0004), with no other significant changes in the remaining thicknesses. In contrast, in the subgroup with no BCVA improvement, a significant increase was reported only in the PR + RPE thickness (from 62.52 ± 3.28 µm to 63.83 ± 3.37 µm, *P* = 0.022), whereas ONL did not change significantly (from 98.32 ± 10.55 µm to 100.27 ± 10.06 µm, *P* = 0.0727).

Correlation analysis showed a significant association between baseline postoperative ONL thickness and final BCVA at 12 months (*r* = 0.45, *P* = 0002) and PR + RPE (*r* = 0.39, *P* = 0.005).

**Figure 2.**
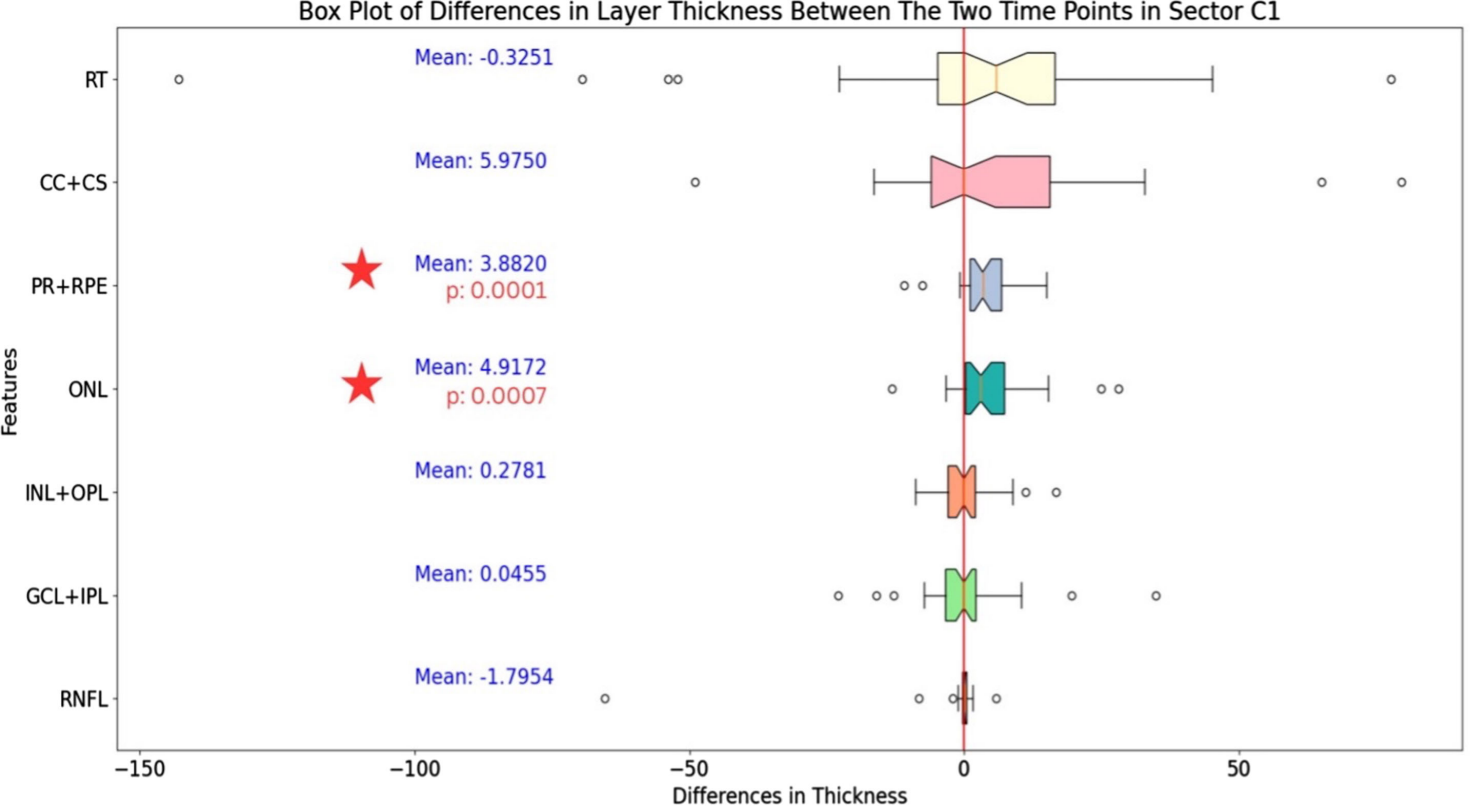
Boxplot representing the variations of different retinal and choroidal thicknesses during the follow-up in the study population.

Volumetric analysis revealed that there were no average significant variations after the follow-up period in IRF (from 6.5 ± 6.8 nL to 2.4 ± 2.9 nL, *P* = 0.1269) and SRF (from 9.6 ± 13.2 nL to 9.6 ± 15.0 nL, *P* = 0.9995) (Table 3, Fig. 2).

**Table 3.** Mean Variations Between First Postoperative and Last Follow-up Visits in Retinal and Choroidal Thicknesses and Retinal Fluids in the Subgroups in Relation to BCVA

Biomarker	First Postoperative Visit	End	Difference of Mean	*P* Value	Subgroup in Relation to BCVA
RNFL (µm)	24.35 ± 5.59	21.59 ± 1.16	2.76	0.3277	Visual improvement
GCL + IPL (µm)	60.97 ± 7.16	61.79 ± 7.81	−0.81	0.7151	
INL + OPL (µm)	53.03 ± 4.64	54.07 ± 5.01	−1.04	0.3965	
ONL (µm)	93.71 ± 6.42	99.99 ± 6.31	−6.28	**0.0022**	
PR + RPE (µm)	61.36 ± 2.60	66.42 ± 2.41	−5.06	**0.0004**	
CC + CS (µm)	202.66 ± 17.84	209.59 ± 22.83	−6.93	0.2153	
RT (µm)	323.59 ± 22.38	324.04 ± 20.68	−0.46	0.9585	
VOL IRF (nL)	11.02 ± 10.99	6.83 ± 8.57	4.2	0.4703	
VOL SRF (nL)	108.06 ± 127.59	44.53 ± 64.85	63.53	0.2592	
RNFL (µm)	22.42 ± 2.47	22.73 ± 2.55	−0.31	**0.0046**	No visual improvement
GCL + IPL (µm)	65.94 ± 13.35	64.30 ± 12.51	1.63	0.3022	
INL + OPL (µm)	62.01 ± 11.21	60.62 ± 10.35	1.39	0.235	
ONL (µm)	98.32 ± 10.55	100.27 ± 10.06	−1.95	0.0727	
PR + RPE (µm)	62.52 ± 3.28	63.83 ± 3.37	−1.31	**0.022**	
CC + CS (µm)	205.97 ± 20.45	209.86 ± 21.63	−3.89	0.0907	
RT (µm)	329.06 ± 43.19	327.03 ± 40.72	2.03	0.7329	
VOL IRF (nL)	25.47 ± 47.55	14.04 ± 23.84	11.44	0.3692	
VOL SRF (nL)	2.97 ± 3.00	3.02 ± 3.35	−0.04	0.9202	

VOL, volume.

Statistically significant findings are presented in bold.

### Biomarker Probability Detection

In the whole cohort, the average occurrence rate of HF was 0.12 ± 0.06 at 4 to 6 weeks postoperatively, and it was reduced to 0.08 ± 0.05 at the last follow-up visit (*P* = 0.0093). The mean occurrence rate of pocket SRF significantly decreased from 0.17 ± 0.09 at 4 to 6 weeks postoperatively to 0.12 ± 0.07 (*P* = 0.0071) at the last follow-up visit.

**Figure 3.**
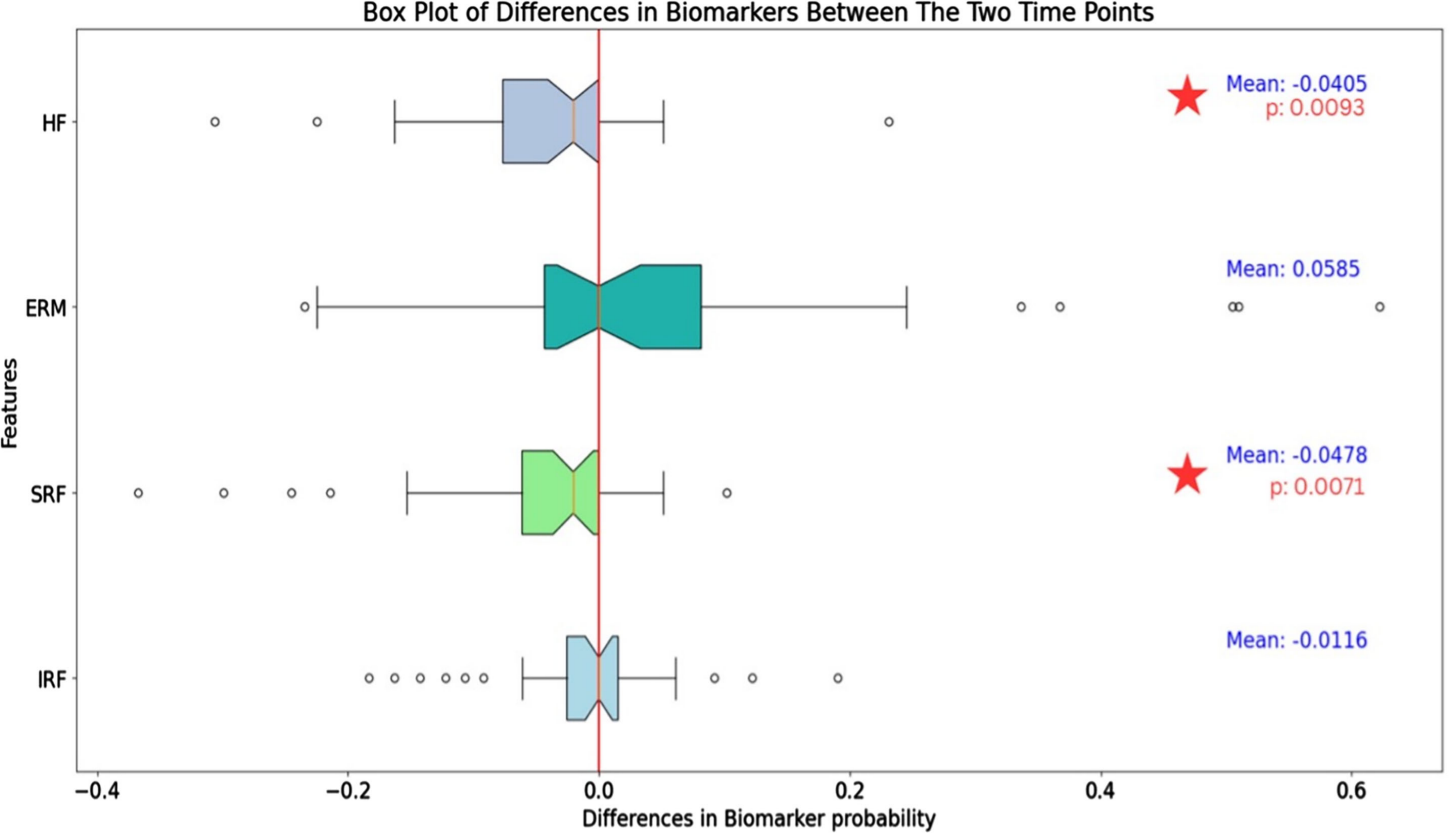
Boxplot representing the variations of biomarker probability during the follow-up in the study population.

No significant changes were detected by the software after the last follow-up visit in the average occurrence rate of IRF and ERM (Table 4, Fig. 3).

**Table 4.** Mean Variations Between First Postoperative and Last Follow-up Visits in AI-Detected OCT Biomarkers in the Subgroups in Relation to BCVA

OCT Biomarker	First Postoperative Visit	Last Postoperative Visit	Difference of Mean	*P* Value	Subgroup in Relation to BCVA
IRF	0.07 ± 0.04	0.05 ± 0.04	0.01	0.3707	Visual improvement
SRF	0.21 ± 0.12	0.13 ± 0.09	0.07	**0.0021**	
ERM	0.41 ± 0.14	0.51 ± 0.14	−0.09	0.0537	
HF	0.13 ± 0.08	0.07 ± 0.06	0.06	**0.0009**	
IRF	0.13 ± 0.09	0.12 ± 0.09	0.01	0.7467	No visual improvement
SRF	0.10 ± 0.10	0.11 ± 0.11	−0.01	0.4198	
ERM	0.44 ± 0.23	0.42 ± 0.23	0.02	0.1031	
HF	0.10 ± 0.06	0.11 ± 0.09	−0.01	0.752	

Statistically significant findings are presented in bold.

In the subgroup with improvement in BCVA, mean occurrence rate of HF significantly decreased from 0.13 ± 0.08 to 0.07 ± 0.06 (*P* = 0.0009) and, similarly, SRF from 0.21 ± 0.12 to 0.13 ± 0.09 (*P* = 0.0021). ERM was found to increase from 0.41 ± 0.14 to 0.51 ± 0.14, but the change was not significant (*P* = 0.0537). Likewise, IRF occurrence rate decreased from 0.07 ± 0.04 to 0.05 ± 0.04 (*P* = 0.3707). In the subgroup with neutral BCVA, no significant changes in biomarker (IRF, SRF, ERM, HF) occurrence rate were reported.

### Regression Models

**Figure 4.**
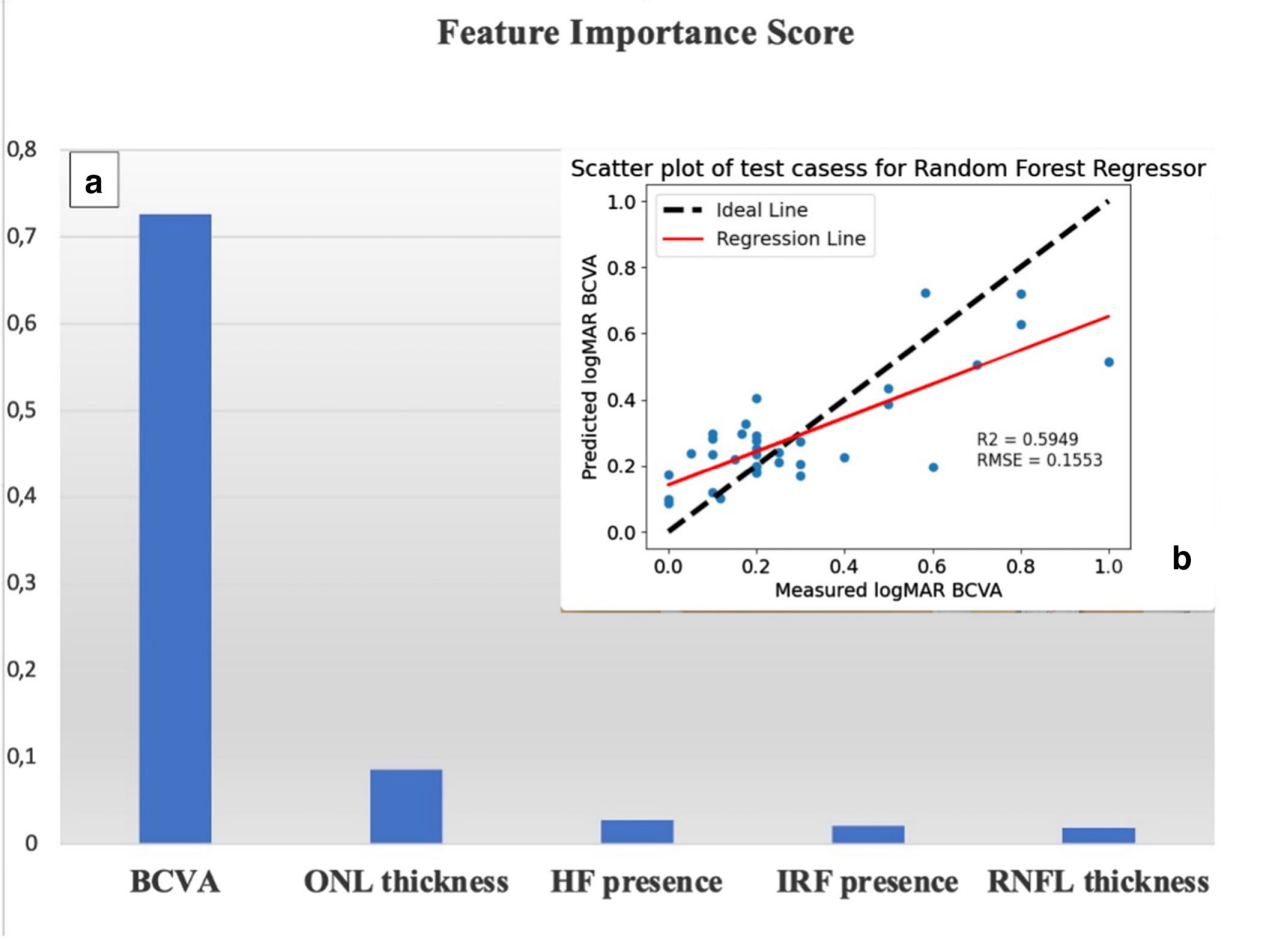
(**a**) Five most important features identified at first visit predictive of final visual acuity in the study group in the random forest model in patients with macula-off retinal detachment. (**b**) Scatterplot of test cases for the random forest model with *r*^2^ and root mean square error. The *red line* represents the regression line, the *dashed black line* shows the ideal linear regression line, and the *dots* indicate the values of the study sample.

The *r*^2^ value of the random forest model was 0.6595. Among the features predictive of the final BCVA, the most influential was the first postoperative BCVA after 4 to 6 weeks of follow-up (FIS = 0.73). The following important features were the distance by baseline ONL thickness (FIS = 0.09), the baseline presence of HF (FIS = 0.03), and the baseline presence of IRF (FIS = 0.02). In Table 5, all the features predictive for final BCVA have been ranked for relative FIS (Fig. 4, Table 5). Moreover, univariate and multivariate analyses are provided in Table 6.

**Table 5.** Relative Feature Importance for Predicting Final BCVA after 12 Months in Patients Operated for Macula-Off Rhegmatogenous Retinal Detachment in the Random Forest Model

Baseline Clinical and Imaging Feature	FIS
BCVA	0.726457
ONL thickness	0.086309
HF biomarker presence	0.028469
IRF_biomarker presence	0.020899
RNFL_thickness	0.019454
ERM_biomarker presence	0.019358
RT_thickness	0.018408
SRF biomarker presence	0.017336
PR + RPE_thickness	0.016202
CC + CS_thickness	0.011708
INL + OPL_thickness	0.01066
IRF_volume	0.009522
SRF_volume	0.008742
GCL + IPL_thickness	0.006477

**Table 6.** Univariate and Multivariate Analysis of the Features Predicting the Final BCVA

Predictor Variable	Univariate Analysis Coefficient (β)	Standard Error (Univariate)	*P* Value (Univariate)	Multivariate Analysis Coefficient (β)	Standard Error (Multivariate)	*P* Value (Multivariate)
First postoperative BCVA	0.73	0.05	<0.0001	0.68	0.06	<0.0001
Baseline ONL thickness	0.09	0.02	0.0012	0.08	0.03	0.0045
Baseline presence of HF	0.03	0.01	0.015	0.03	0.02	0.021
Baseline presence of IRF	0.02	0.01	0.045	0.02	0.01	0.047
Change in PR + RPE thickness	0.12	0.04	0.003	0.10	0.05	0.00

## Discussion

We demonstrated that a thicker ONL and PR + RPE complex at 4 to 6 weeks postoperatively were positively linked to better visual outcomes. We also reported that the presence of HF at the first follow-up visit was associated with poorer visual outcomes in these patients. Additionally, we emphasized the predictive significance of initial BCVA and, to a lesser extent, IRF presence for final visual outcomes.

Several previous studies have highlighted the presence of different OCT biomarkers descriptive of the outer nuclear layer status in association with visual prognosis.^8^ Among them, the EZ-RPE thickness, INL + OPL and ONL thicknesses, the inner segment/outer segment (IS/OS) distortion, and the integrity of ELM have been identified as OCT biomarkers predictive for final BCVA.^11^^,^^19^^–^^21^ Delolme et al.^22^ found the presence of microstructural modifications at the level of PR in 66.7% of the subjects and a disruption in IS/OS segments in 53.3% of the subjects in the study population with macula-off RRD. Menke et al.^23^ found that in patients with successful macula-off RRD, there was a positive correlation between an increase in INL + OPL and EZ-RPE thicknesses and time after surgery; however, no significant correlation with final postoperative BCVA could be extrapolated in this study. Similarly, in our study, we found that the significant increase in ONL and PR + RPE thicknesses in the follow-up period was positively correlated with better visual outcomes in the study population. This is in accordance with the findings described in different animal models where outer segments may take a very long time to recover their predetachment length.^24^

In experiments measuring the regrowth of rod outer segments in the cat retina, it was found that even after a month of reattachment, they had not regained their original length.^24^ Similarly, research by Guerín et al.^25^ on rhesus macaques showed that both rods and cones recovered less than 50% of their length after 30 days. It took 150 days for these segments to reach lengths comparable to those in normal animals, although the length distribution still varied, with many cones only reaching about two-thirds of the control group's length; however, in some cases, the EZ-RPE may not be completely restored even after 1 year, and this could be predicted by the presence of pronounced thinner ONL and PR + RPE at 4 to 6 weeks postoperatively, indicating photoreceptor cell death, as demonstrated by our multivariate analysis describing a negative correlation between a lower thickness at baseline and less favorable visual outcomes (logMAR) after the follow-up visit.

Regarding the HF, we reported a significant reduction of them from first to last follow-up visit, indicating a potential association with improved final BCVA. The role of HF in the postoperative period after RRD repair has been investigated by some studies. A recent study published by Wu et al.^26^ calculated manually the changes of HF in RRD after successful reattachment surgery in 29 patients and evaluated the relation between HF presence and PR status during the follow-up. The authors described an increase of HF incidence in the outer retina in the first 3 months after surgery, followed by their subsequent decline after 3 months. HF was mainly localized to areas where ELM and IS/OS were disrupted; in these cases, a higher number of HF were exhibited in comparison with eyes with a continuous IS/OS line. Regarding BCVA, the authors reported that the number of HF at 0.5 months was positively linked to favorable visual outcomes, while their presence at 3 months was, by contrast, associated with poorer visual function. The authors postulated that HF activation was related to inflammatory activity in response to PR damage, with an increase of HF when the IS/OS was disrupted and a subsequent decrease upon repair. Similarly, Jhaveri et al.^27^ found that hyperreflective dots were associated with the increasing stage of RRD, reduced integrity of foveal photoreceptors, and decreased postoperative visual acuity at 3, 6, and 12 months. In our study, we described that a higher occurrence of HF within 4 to 6 weeks from surgery was a moderate predictor of poorer visual outcomes, with a subsequent HF reduction found at last baseline visit (between 6 and 12 months) associated with an improvement of vision.

Our findings are also in line with previous studies on other retinal diseases, including age-related macular degeneration (AMD), diabetic macular edema, and retinal vein occlusions, providing evidence of worse visual prognosis associated with the presence of HF.^28^^–^^30^ A recent study correlating OCT imaging and immune-histochemical analysis of donor AMD eyes postulated the possible origin of HF as transdifferentiated RPE cells, which underwent an epithelial–mesenchyme transition due to an underlying presence of a proinflammatory drive intrinsic to the disease.^31^ In this regard, previous studies on various vascular diseases have indicated that HF could be activated RPE cells differentiated into microglial cells in response to various proinflammatory stimuli.^32^ In animal models of RRD, it was observed that activated microglia rapidly migrated to the damaged PR layer to engulf the injured or dying cells, and the depletion of microglia was associated with increased PR cell death.^33^ In RRD, the subretinal space is usually free of cells; however, within 24 hours of retinal detachment, a number of cell types (polymorphonuclear neutrophils, monocytes, and macrophages) migrate into this space from the choroidal and retinal capillaries. Free RPE cells are also seen in the subretinal space within 72 hours of retinal detachment and frequently contain outer-segment fragments, indicating that they may play a role in phagocytosis of cellular debris.^34^^,^^35^ Considering our findings in conjunction with previous preclinical studies, we hypothesize that HF may not only serve as biomarkers for disease activity, particularly microglial activation triggered by the inflammatory mechanism following macular-off RRD and its surgical repair, but also play a predictive role in establishing visual function.

In line with previous studies, we showed that BCVA at the first postoperative visit was the strongest predictor of 12-month vision outcomes following macula-off RRD repair^36^^,^^37^; however, we suggest that further studies with longer follow-up periods are needed to better describe this relationship. Moreover, we found that baseline postoperative presence of IRF, which was identified in 22% of the patients, was also a moderate predictor of final BCVA.

We acknowledge that our study has some limitations, among them the retrospective nature of the study and the relatively small sample size of the patients. Despite the limitations, our study sheds light on the use of AI for OCT biomarker identification in macula-off RRD. Key findings, such as the positive link between thicker ONL and PR + RPE at 4 to 6 weeks postoperatively and improved visual outcomes, along with reduced HF presence in the subgroup of patients with better final BCVA, contribute to the field. Our study showed the potential application of AI in vitreoretinal disorders, emphasizing early OCT biomarkers’ relevance in predicting 1-year postoperative visual acuity.

## Conclusions

This study using AI software in macula-off RRD found a positive association between thicker ONL and PR + RPE at the first postoperative visit, correlating with improved visual outcomes. The relatively novel finding that HF presence at first postoperative visit predicts poorer outcomes aligns with broader evidence in retinal diseases, requiring further histologic validation. Baseline BCVA proved the most predictive for final functional outcomes in macula-off RRD patients. All these clinical features may help in the prognostication of functional outcomes in patients with macula-off RRD.
